# Ozone/graphene oxide catalytic oxidation: a novel method to degrade emerging organic contaminant *N, N*-diethyl-*m*-toluamide (DEET)

**DOI:** 10.1038/srep31405

**Published:** 2016-08-11

**Authors:** Jia-Nan Liu, Zhuo Chen, Qian-Yuan Wu, Ang Li, Hong-Ying Hu, Cheng Yang

**Affiliations:** 1 Environmental Simulation and Pollution Control State Key Joint Laboratory, State Environmental Protection Key Laboratory of Microorganism Application and Risk Control (SMARC), School of Environment, Tsinghua University, Beijing 100084, P. R. China; 2 Shenzhen Laboratory of Microorganism Application and Risk Control, Graduate School at Shenzhen, Tsinghua University, Shenzhen 518055, P. R. China; 3 Shenzhen Environmental Science and New Energy Technology Engineering Laboratory, Tsinghua-Berkeley Shenzhen Institute, Shenzhen 518055, P. R. China; 4 Division of Energy and Environment, Graduate School at Shenzhen, Tsinghua University, Shenzhen 518055, P. R. China

## Abstract

*N, N*-diethyl-*m*-toluamide (DEET) is one of the important emerging contaminants that are being increasingly detected in reclaimed water as well as in drinking water sources. However, DEET is refractory to conventional biological treatment and pure ozone which is absent of hydroxyl radical. Current researches on the efficient removal of DEET are still quite limited. This study utilizes a novel method, namely ozone/graphene oxide (O_3_/GO), to investigate the effects on DEET removal in aqueous systems, especially in reclaimed water. The results indicate that the DEET degradation rate was significantly accelerated through the combined effect of GO and ozonation which can yield abundant hydroxyl radical, compared to pure ozone condition. According to hydroxyl radical scavenging experiments, hydroxyl radical was found to play a dominant role in synergistic removal of DEET. These findings can offer sound suggestions for future research on the removal of emerging organic contaminants. The information could also be beneficial to reclaimed water safety and sustainable management.

*N, N*-diethyl-*m*-toluamide (DEET) has been widely used as an insect repellent substance to prevent arthropod biting in our daily life over the last 60 years in terms of several formulations (e.g., lotions, gels, mists, aerosols and sticks)^1^,^2^. Due to its persistence and toxicity, DEET is considered as an emerging organic contaminant that has been increasingly detected in aquatic environments worldwide, such as reclaimed water, surface water, seawater and even in drinking water^3^,^4^,^5^. The concentration range of DEET in aqueous environment is about ng/L to μg/L^6^.

For instance, Sun *et al*.^7^ have detected the DEET in drinking water sources of five major river watersheds in China. Researches have shown that DEET has potential genotoxic properties in human nasal mucosal cells^8^. Other studies have reported the neurotoxicity of DEET to insects and mammals^9^ which can cause child coma and seizures after ingestion of low doses (80 mg/kg)^10^. Besides, a significant reduction of carbohydrates levels of *Sericostoma vittatum* after exposure to DEET was also observed^11^. Consequently, it is vital and urgent to control the concentration of DEET in reclaimed water and other aquatic environment. Current studies verify that DEET is resistant to biodegradation processes in wastewater treatment plant^12^,^13^. Membrane bioreactors (MBRs) also show variable removal efficiencies of DEET^14^. Acero *et al*.^15^ studied the elimination of emerging contaminants (ECs) in three selected water matrices and indicated that DEET is refractory to pure ozone, and the presence of H_2_O_2_ in addition to ozone can enhance the DEET removal due to the production of hydroxyl radical (∙OH). The second-order reaction rate constant with ozone and ∙OH at pH 7 is (0.126 ± 0.006) M^−1^∙s^−1^ and (4.95 ± 0.18) × 10^9^ M^−1^∙s^−1^, respectively^16^,^17^. Therefore, oxidation via hydroxyl radical can be an effective way to eliminate DEET.

In recent years, catalytic ozonation is considered as an effective way to abate refractory organic compounds for improving the concentration of ∙OH. Many studies have highlighted the use of carbon material, on account of its dual role both as adsorbent and as catalyst which can interact with ozone simultaneously, leading to hydroxyl radical generation^18^,^19^,^20^,^21^. The mechanism behind could be the interaction between ozone and pyrrol group or the oxygen containing functional groups^18^,^19^,^20^. In this study, it is found that the surface of graphene oxide (GO) contains multiple oxygen-containing groups, particularly hydroxyl and carboxyl^22^,^23^,^24^. Moreover, GO can be easily obtained from natural graphite using the modified method developed by Hummers^25^, achieving functionalized self-assembled on metal oxide (ZnO, MnO_2_ nanowires)^26^,^27^ and converting oxygen contents when varying the thermal annealing temperature^28^, even ozone could re-oxidize graphene oxide forming O-GO, which contains more carbonyl and carboxy improving catalytic active of GO^29^. It’s reported^24^ that reduced graphene oxide (rGO), the reduction state of GO, exhibited a superior activity in activating ozone for catalytic oxidation of organic p-Hydroxylbenzoic Acid (PHBA) and carbonyl groups were identified as the active sites for the catalytic reaction. But according to the XPS spectra on C 1s, the carbonyl groups content of GO (41.61%) was significantly higher than rGO (5.69%). While the enhancement of GO on formation of ∙OH during ozonation remained unknown. Despite there are considerable literature studying on GO adsorption property so as to remove both inorganic and organic contaminants in the environment^22^,^30^, seldom research uses it as catalyst coupling with ozone to degrade organic contaminants and illuminate the mechanism. Therefore, in this paper, GO is applied as a new catalyst to explore its catalytic characteristics.

The aim of this study is to analyse the synergistic degradation of DEET during ozone/GO catalytic oxidation and to evaluate the contribution of hydroxyl radical on DEET removal. Furthermore, the catalytic ability of GO during ozonation at different conditions such as the variations of GO dose, pH, matrices in reclaimed water were also evaluated.

## Results and Discussion

### Degradation of DEET during ozone/GO

**Figure 1:**
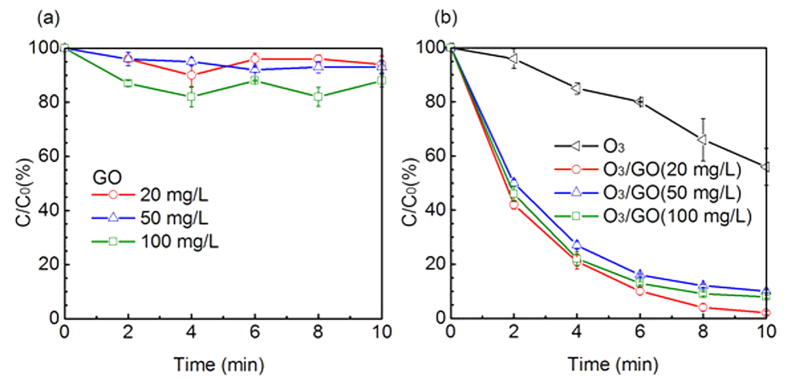
Normalized DEET concentration as a function of time containing different concentration of GO for (**a**) adsorption and (**b**) catalytic ozonation (O_3_/GO). Experimental conditions: [DEET] = 50 μM, [PBS] = 10 mM, pH = 7.

**Figure 2:**
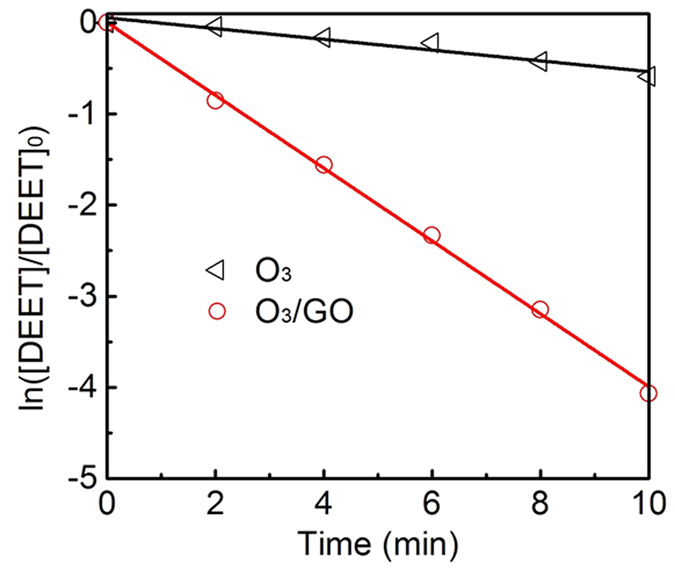
The pseudo-first-order reaction plots of O_3_ and O_3_/GO processes. Experimental conditions: [DEET] = 50 μM, [GO] = 20 mg/L, [PBS] = 10 mM, pH = 7.

Changes of DEET concentration during single ozonation effect, single GO adsorption effect and ozone/GO combination effect are shown in Fig. 1. Carbon material can simultaneously act as an adsorbent and a catalyst during ozonation process. The removal of DEET during single GO adsorption at different GO concentrations was very limited, indicating that using GO as an adsorbent for adsorption removal of DEET was unfeasible. Comparatively, during O_3_/GO oxidation process (Fig. 1b), DEET concentration was declined dramatically and an over 95% of removal efficiency was achieved within 10 mins, while the sole ozonation process only contributed to approximately 40% of DEET removal. Furthermore, the pseudo-first-order rate constant of O_3_/GO ((0.399 ± 0.007) min^−1^) was almost six times higher than single ozonation ((0.059 ± 0.006) min^−1^) (Fig. 2). According to the results of adsorption and catalytic ozonation experiments in this study, the decay of DEET was depended on the oxidation of DEET, unlike the previously reported multiwalled carbon nanotubes/iron oxides which adsorbed 1-naphthol on the catalyst, then oxidized it by ozone^31^. Beltran *et al*.^32^ discovered that the removal of pyruvic acid by O_3_/AC achieved a removal efficiency of 90%, compared to approximately 10% by sole ozonation effect. Aside from AC, MWCNTs synergized with ozone can also enhance the degradation of refractory organic pollutants compared to sole ozonation^20^.

When increasing the GO concentrations, the catalytic efficiency of O_3_/GO was not enhanced as much as expected. This indicates that it is not an effective way to promote the conversion of O_3_ into ∙OH by simple addition of GO. An analogous phenomenon of catalytic ozonation of ciprofloxacin with MnO_2_ was also observed^33^. Beltran *et al*.^34^ reported that the oxidation rate slightly decayed when the catalyst amount was above the optimal value during catalytic ozonation. The similar catalytic efficiency at different GO doses indicated that GO powder may block the ozone mass transfer process or excess GO may quench the active species, such as ∙OH. Sanchez-Polo *et al*.^35^ found that there was a slight difference of the k_D_ value between O_3_/H_2_O_2_ and O_3_/GAC, while the R_ct_ values, defined as the ratio of ∙OH exposure to O_3_ exposure, were different.

### Effects of free-radicals on DEET removal

**Figure 3:**
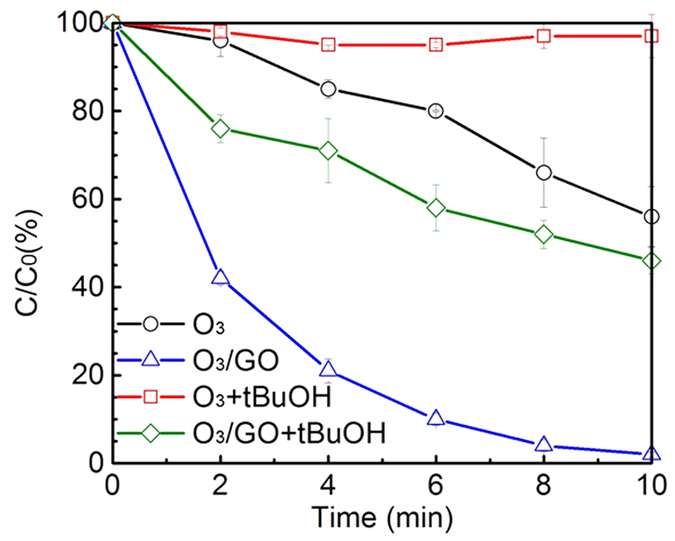
Normalized DEET concentration as a function of time in the presence of tBuOH. Experimental conditions: [DEET] = 50 μM, [GO] = 20 mg/L, [PBS] = 10 mM, [tBuOH] = 320 μM, pH = 7.

**Figure 4:**
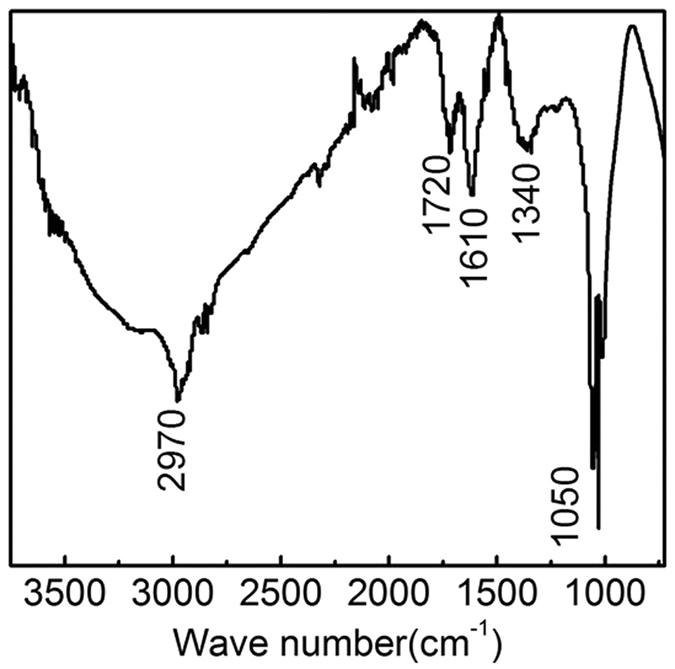
FTIR spectra of the GO.

Tert-butyl alcohol (tBuOH), a kind of hydroxyl radical scavenger (k_∙OH, tBuOH_ 6 × 10^8 ^M^−1^∙s^−1^ 36), was used to evaluate the contribution of hydroxyl radical on the oxidation of DEET and the results were shown in Fig. 3. It can be observed that the removal rate of DEET during ozone or ozone/GO process in the presence of tBuOH was much lower than that without the addition of tBuOH. The sole ozonation in the presence of tBuOH scavenging hydroxyl radical could hardly contribute to the degradation of DEET. This indicated that the contribution of pure ozone to the removal of DEET was quite limited; however, ozone in conjuction with GO is capable of achieving high removal efficiency (nearly 50% of DEET removal) when the majority of hydroxyl radical was quenched. The phenomenon is in good agreement to the result reported by Rebekah *et al*.^20^, in which the reaction parameters are similar to this study except for ozone usage. Tay *et al*.^37^ added tBuOH to scavenge ∙OH and confirmed that DEET was resistant against ozone degradation. Guo *et al*.^38^ demonstrated that MnO_2_-Co_3_O_4_ could promote the production of ∙OH in ozonation process. Those studies imply that the O_3_/GO oxidation process could generate active species ∙OH which is a key factor for DEET reduction. In addition, Fig. 4 shows that GO contains multiple oxygen-containing groups which can interact with ozone yield a mass of hydroxyl radical. The spectrum of GO exhibits a -OH stretching vibration at 2970 cm^−1^, a −C=O stretching vibration at 1720 cm^−1^, a C=C stretching vibration at 1610 cm^−1^, a -COOH stretching vibration at 1340 cm^−1^ and a -C-O stretching vibration at 1050 cm^−1^.

**Figure 5:**
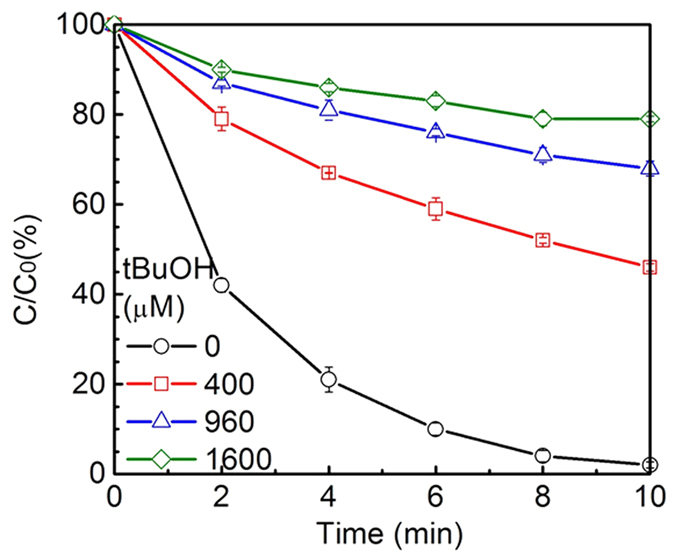
Normalized DEET concentration as a function of time during ozonation of GO containing different concentration of tBuOH. Experiment conditions: [DEET] = 50 μM, [GO] = 20 mg/L, [PBS] = 10 mM, pH = 7.

To further analyze the effectiveness of sole O_3_/GO on DEET removal without the contributing effect of ∙OH, tBuOH concentration was increased from 400 μM to 1600 μM. (corresponding to the molar ratio of [tBuOH]/[DEET] increased from 8 to 32) and the results are shown in Fig. 5. The degradation rate of DEET was slowed down in accompany with the reduction of the amount of hydroxyl radical. The removal efficiency of DEET during ozone/GO decreased to 20% in the presence of 1600 μM tBuOH. This phenomenon indicates that GO is an effective catalyst when synergizing with ozone for an advanced oxidation process to decompose refractory organic pollutants.

### Effects of pH on DEET removal

**Figure 6:**
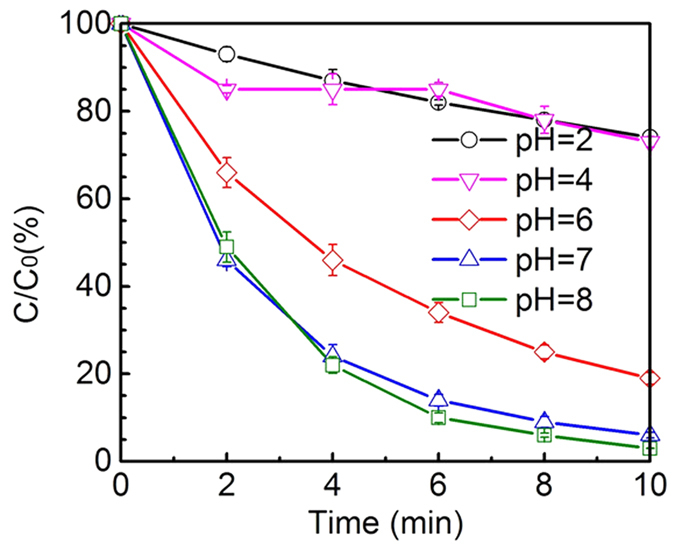
Normalized DEET concentration as a function of time during ozonation of GO at various pH values. Experiment conditions: [DEET] = 50 μM, [GO] = 20 mg/L.

Organic matters in solutions with various pH values present diverse dissociative states. Non-dissociating and dissociating contaminants possess different structural characteristics when reacting with O_3_ or ∙OH in unique rate constants^39^. On the other hand, pH can change the decomposition rate of ozone molecule^38^. Therefore, it’s necessary to elucidate the influence of pH on DEET removal during O_3_/GO catalytic oxidation. The degradation status of DEET by O_3_/GO at different pH conditions is shown in Fig. 6. It can be seen that the elimination of DEET was significantly inhibited at acidic conditions. The DEET loss was decreased from 95% to 20% when pH was adjusted from 7 to 2~4, respectively. Moreover, the tendency of inhibition was more intensive at lower pH levels. The removal rate of DEET at alkaline condition (pH 8) was similar to that of the neutral aqueous condition. Since the pK_a_ value of DEET is 0.4^16^, the effect of DEET form is negligible. Theoretically, OH^−^ ion can enhance ozone molecules transform into ∙OH radicals, so that the decay rate of the target contaminants could be largely accelerated with the increase of pH values^40^. However, in real situations, when pH is increased from 7 to 8, the promoting effect is not remarkable. As observed by Tay *et al*.^41^, under buffered condition, the k_obs2_ of DEET is increased when rising pH, then is decreased when the pH value exceeds the threshold. Possible reasons may be that high concentration OH^−^ ion leads to ozone instability in aqueous solution, resulting in a reduction of dissolved O_3_^42^.

### Effect of humic acid on DEET removal

**Figure 7:**
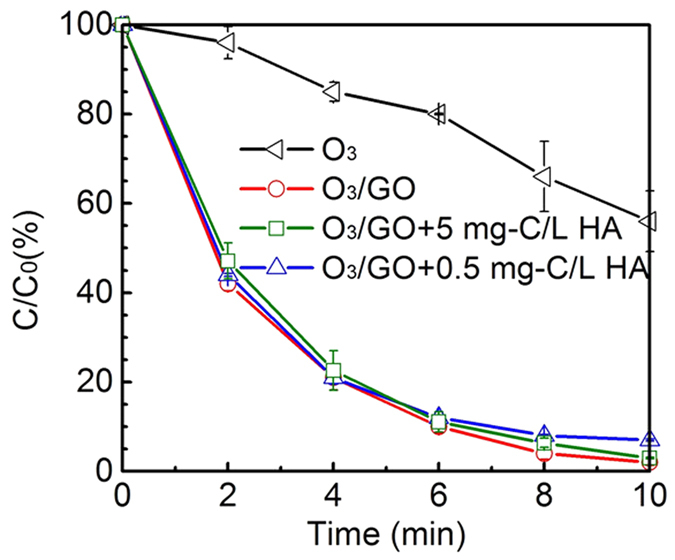
Normalized DEET concentration as a function of time in the presence of HA. Experiment conditions: [DEET] = 50 μM, [GO] = 20 mg/L, [PBS] = 10 mM, pH = 7.

Reclaimed water is a complex system with various compositions, including organics, inorganic salts and nutrients. There are numerous dissolved organic matters (DOM) in reclaimed water which can react with ozone either directly or indirectly. Thus, humic acid (HA), a fundamental component of natural organic matter, can be used as a surrogate to investigate the interaction between DOM and O_3_/GO system. As shown in Fig. 7, the addition of HA (0.5 mg-C/L and 5 mg-C/L) led to a non-obvious variation but only a slight inhibition of the removal of DEET. Even if the addition of HA up to 5 mg-C/L, the efficiency of the process based on the combined O_3_/GO system was not influenced. This result is in a good accordance with the experimental results obtained by Rebekah *et al*.^20^ where the HA just slightly impacted on the degradation of organic matters during catalytic ozonation when coupling MWCNTs with ozonation. Nevertheless, it was reported that ∙OH radical is a predominant type of active species to remove humic acid in water^43^, while the addition of isopropanol causes the HA loss decreased by nearly half. To better explain the complex reactions, the rates and concentrations of HA and DEET used in the experiment were compared. Notably, the reaction rate of HA and ∙OH is (1.4 ± 0.2) × 10^4^ L∙mg-C^−1^∙s^−1^ 44, which is five orders of magnitude lower than that of DEET ((4.95 ± 0.18) × 10^9 ^M^−1^∙s^−1^ 17), and the concentration of HA is higher than DEET ([HA]/[DEET] = 10^4^~10^5^ mg-C/mol). taken these two factors into consideration,the reaction competitivenessof DEET is 3.5~35 times higher than HA, which can cause the slightly inhibition in the presence of HA during the O_3_/GO catalytic ozonation. Ikhlaq *et al*.^45^,^46^ profoundly evaluated the effect of humic acid on ibuprofen and cumene degradation during catalytic ozonation and concluded that if the catalyst showed a low adsorption capacity towards humic acid, the catalytic activity is not influenced by humic acid; whereas, humic acid can inhibit the catalyst generating hydroxyl radicals. Therefore, it is reasonable to conjecture that GO is difficult to adsorb of humic acid on its surface.

### Effects of bicarbonate on DEET removal

**Figure 8:**
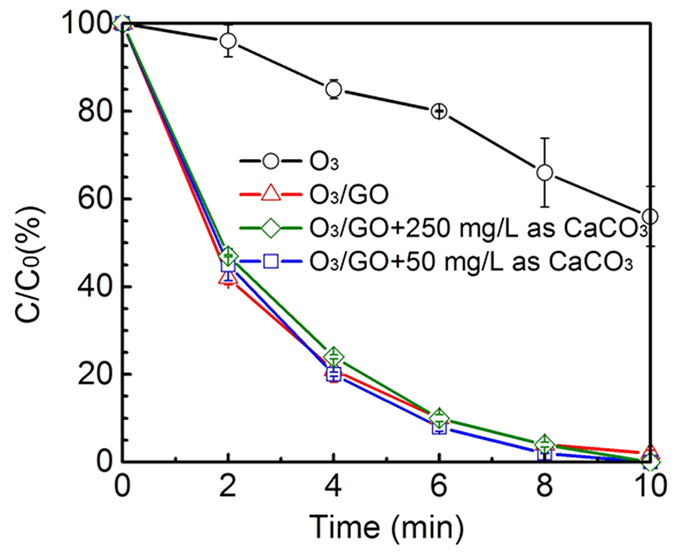
Normalized DEET concentration as a function of time in the presence of HCO_3_^−^. Experiment conditions: [DEET] = 50 μM, [GO] = 20 mg/L, [PBS] = 10 mM, pH = 7.

Carbonate (CO_3_^2−^) and bicarbonate (HCO_3_^−^), another unneglectable matrices, as natural inhibitors in reclaimed water, can react with ∙OH with second rate constants of 3.9 × 10^8^ M^−1^∙s^−1^ and 8.5 × 10^6^ M^−1^∙s^−1^ 36. In neutral solution, inorganic carbon is prone to form bicarbonate ion^47^, and thus the proportion of HCO_3_^−^ is a dominant inorganic carbon in neutral solution. As can be seen from Fig. 8, the removal rate of DEET in the presence of bicarbonate was slightly lower than the one in the absence of bicarbonate. Additionally, the ozonation removal of DEET was not notably changed with the increase of carbonate dose. The slight discrepancy between the presence and absence of HCO_3_^−^ was also experimentally observed by Rebekah *et al*.^20^ and Bai *et al*.^48^ utilizing the approaches of O_3_/MWCNTs and O_3_/Fe_3_O_4_/MWCNTs, respectively. From a theoretical standpoint, the degradation of target contaminant primarily according to hydroxyl radicals should decelerate with the addition of HCO_3_^−^ which acts as hydroxyl radical scavenger. Zhao *et al*.^49^ discovered that the removal rate of nitrobenzene (an indicator of radical ∙OH) increased first and decreased subsequently with the bicarbonate concentration increased from 0 to 250 mg/L in either the process of ozone/ceramic honeycomb or ozone/Mn-ceramic honeycomb. The reason why the presence of HCO_3_^−^ just caused a little inhibition is because the reaction rate of HCO_3_^−^ with ∙OH is 8.5 × 10^6^ M^−1^∙s^−1^, which is three orders of magnitude lower than DEET ((4.95 ± 0.18) × 10^9^ M^−1^∙s^−1^ 17) and the content of HCO_3_^−^ is only slightly higher than DEET ([HCO_3_^−^]/[DEET] = 10~50 mol/mol).

### Effects of matrices in reclaimed water on DEET removal

**Figure 9:**
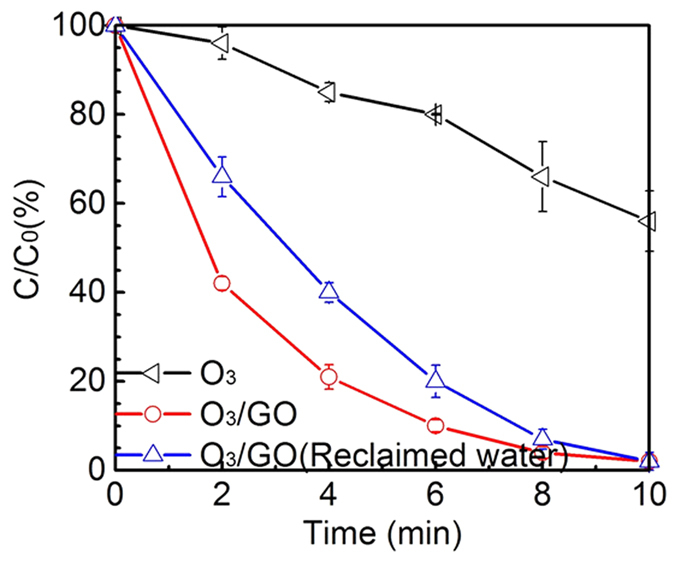
Normalized DEET concentration as a function of time in reclaimed water. Experiment conditions: [DEET] = 50 μM, [GO] = 20 mg/L, [PBS] = 10 mM, pH = 7.

Ultimately, catalytic ozonation experiment was carried out in reclaimed water to verify the feasibility of O_3_/GO oxidation system in real environment. The reclaimed water was spiked with DEET. For comparison, ultrapure water with DEET was conducted synchronously under the same reaction condition. Figure 9 depicts that the presence of reclaimed water matrices can suppress the elimination of DEET during O_3_/GO catalytic oxidation process. It is worth to note that even there are various complex matrices in reclaimed water, the effect of catalytic activity is also appreciable comparing the two different water quality conditions. Therefore, a considerable synergistic effect of graphene oxide and ozone forming hydroxyl radicals can contribute to the degradation of DEET. Similar results were found by Jin *et al*.^39^ who tested reaction kinetics of twenty-four micropollutants with diverse chemical structure in ozonation and advanced oxidation processes (O_3_/H_2_O_2_ or UV/H_2_O_2_) and corroborated that the application of advanced oxidation depended on hydroxyl radicals is an effective technology to degrade target contaminants. The phenomenon is based on the property of nonselective hydroxyl radicals.

## Conclusions

Insect repellents such as DEET are widely found in reclaimed water and other freshwater systems. Increasing concern has been paid to their ecological toxicity and other chronic effects on aquatic environment. However, current knowledge on the efficient removal of DEET is still scarce. This study demonstrated that ozone/GO can be considered as a novel and promising method that has shown remarkable performance regarding DEET degradation and removal. Particularly, the combination of GO and ozonation can significantly accelerate DEET degradation, while adsorption of DEET on GO is limited. This phenomenon could be explained by the fact that during ozone/GO, hydroxyl radical is formed and resulted in synergistic removal of DEET. We also evaluated the elimination of DEET during ozone/GO was in complex conditions. The removal of DEET is significantly inhibited at acidic conditions, suggesting that hydroxyl radical is quenched at lower pH levels. Matrices in reclaimed water can inhibit the degradation of DEET, while the effects of HCO_3_^−^ and humic acids on DEET removal are limited. Overall, this study indicated that the novel GO and ozonation combined approach can be considered as a viable strategy for the removal of emerging organic contaminants in reclaimed water. In the future, more work will be conducted on oxidation byproducts of DEET and determining the mineralization rate by TOC analysis during catalytic ozonation via GO.

## Materials and Methods

### Chemicals and materials

Graphene oxide (concentration: 2 g/L, purity: >98.5 wt%, O: 46–49 wt%, S: <1.0 wt%, K^+^: <0.15 wt%, Mn^2+^: <0.01 wt%) was obtained from Carmery Materials Technology Co., Ltd. (Taiyuan Shanxi). DEET (purity: >98%) was purchased from J&K Scientific, Ltd. (China). Methanol of HPLC grade was provided by J.T. Baker (Avantor, China). Tert-butyl alcohol (tBuOH), humic acid (HA), disodium hydrogen phosphate, monosodium phosphate and sodium bicarbonate were of analytical grade and were all used as received without any further purification. All solutions were prepared using ultrapure water (UPW) from a Milli-Q purification system (Integral 5, Millipore, Unites States).

Reclaimed water was obtained from Xili wastewater treatment plant (WWTP), Shenzhen, China, where the reclaimed water was treated by anaerobic-anoxic-oxic process, coagulation, filtration, and UV disinfection processes. The collected reclaimed water was then filtrated by 0.45 μm glass microfiber filters (Grade GF/F circles, 110 mm, 25/pk, Whatman^TM^, UK) and stored at 4 °C. The dissolved organic carbon (DOC) was analyzed using TOC-VCPH (Shimadzu, Japan). Reclaimed water samples have an average DOC concentration of 3.58 mg/L and UV absorbance of 0.0648 cm^−1^. Phosphate (pH 7, 10mM) was dosing into the water to enhance the buffer capacity^50^.

### Ozonation and adsorption

Ozonation and adsorption were both carried out in 250 mL semi-batch mode at room temperature. Ozone was generated from purified oxygen (99.8%) using O_3_ generator (Ozocenter, China) and analyzed by O_3_ detectors (IDEAL-2000, China). While the concentration of ozone gas reached 5 mg/L maintaining a steady state and the flowing rate kept at 0.4 L-gas/min; ozone was continuously bubbled into reactor through gas dispersion. In the catalytic ozonation experiment, reactions were initiated by introducing ozone into 200 mL DEET aqueous solution (50 μM) containing a certain amount of GO under magnetic stirring at 800 rpm. Samples were periodically withdrawn and filtered by 0.22 μm hydrophilic polyethersulfone (PES) syringe filter (ANPEL Laboratory Technologies, Shanghai). The solution pH was adjusted by phosphoric acid and sodium/potassium phosphate.

### Analytical method and GO characterization

The variation of DEET concentration was monitored by a high performance liquid chromatograph (HPLC, 20 AT, Shimadzu, Japan) equipped with a photo-diode array (PDA, SPD, Shimadzu, Japan). Chromatographic separation was performed using a 5C8-MS column (4.6 mm × 250 mm, COSMOSIL, Nacalai Tesque, Inc., Japan). The mobile phase was a mixture of methanol and ultrapure water (80:20, V/V) at a flow rate of 0.7 mL/min. The detection wavelength was 210 nm. GO was characterized via Fourier Transform Infrared Spectroscopy (FTIR).

## Additional Information

**How to cite this article**: Liu, J.-N. *et al*. Ozone/graphene oxide catalytic oxidation: a novel method to degrade emerging organic contaminant *N, N*-diethyl-*m*-toluamide (DEET). *Sci. Rep.* **6**, 31405; doi: 10.1038/srep31405 (2016).
